# Ultraviolet Irradiation on a Pyrite Surface Improves Triglycine Adsorption

**DOI:** 10.3390/life8040050

**Published:** 2018-10-25

**Authors:** Santos Galvez-Martinez, Eva Mateo-Marti

**Affiliations:** Centro de Astrobiología (CSIC-INTA), Ctra. Ajalvir, Km. 4, 28850 Torrejón de Ardoz, Spain; sgalvez@cab.inta-csic.es

**Keywords:** pyrite, triglycine, XPS, peptide, sulfide mineral, UV, surface, adsorption, prebiotic chemistry

## Abstract

We characterized the adsorption of triglycine molecules on a pyrite surface under several simulated environmental conditions by X-ray photoemission spectroscopy. The triglycine molecular adsorption on a pyrite surface under vacuum conditions (absence of oxygen) shows the presence of two different states for the amine functional group (NH_2_ and NH_3_^+^), therefore two chemical species (anionic and zwitterionic). On the other hand, molecular adsorption from a solution discriminates the NH_2_ as a unique molecular adsorption form, however, the amount adsorbed in this case is higher than under vacuum conditions. Furthermore, molecular adsorption on the mineral surface is even favored if the pyrite surface has been irradiated before the molecular adsorption occurs. Pyrite surface chemistry is highly sensitive to the chemical changes induced by UV irradiation, as XPS analysis shows the presence of Fe_2_O_3_ and Fe_2_SO_4_—like environments on the surface. Surface chemical changes induced by UV help to increase the probability of adsorption of molecular species and their subsequent concentration on the pyrite surface.

## 1. Introduction

Minerals can be very promising surfaces for studying biomolecule surface processes, which are of principal relevance in the origin of life and a source of chemical complexity [1,2]. Bernal proposed that mineral surface adsorption could help to overcome the problem of the extremely dilute concentration of amino acids in prebiotic oceans [3]. Minerals such as silicates, oxides and sulfides were probably present on early Earth in several environments [4,5], and among them, pyrite (FeS_2_) is one of the most important and abundant sulfide minerals on Earth. Indeed, many redox-based geochemical and biogeochemical processes, such as the cycling of S, Fe, and other elements in the environment or in deposit formation, occur on the surface of pyrite [6]. Additionally, due to its catalytic activity, the pyrite surface plays an important role in heterogeneous catalysis [7,8] and a role in the origin of life and prebiotic chemistry [9,10]. Huber and Wächtershäuser proposed the theory of an ‘‘iron–sulfur world’’ [11] in that the first reactions that led to the formation of amino acids occurred on the surface of minerals (such as pyrite) because such surfaces may adsorb and concentrate these biomolecules and catalyse the reactions. It has been proposed that the first reactions that led to the formation of amino acids did not occur in a bulk solution in oceans but on the surface of minerals because such surfaces have the potential to facilitate prebiotic polymerization [12,13,14]. The presence of iron-sulfur cores in several modern proteins, such as ferredoxins, has been offered as additional evidence for the “iron-sulfur” world hypothesis.

Mineral surfaces could potentially allow for almost any type of general catalysis, with low specificity and efficiency. Understanding the possible contribution of mineral surfaces to peptide oligomerization and how minerals could play a relevant role in concentrating biomolecules at the surface and/or providing catalytic sites on the surface is critical for understanding the origin of life [10,11,12,13,14,15].

The adsorption of small molecules onto specific mineral surfaces for selective concentration [16], catalytic polymerization [17], protection from UV light [18], etc., is critical for understanding the selective mechanisms for the accumulation of amino acids or nucleotides involved in chemical evolution. The surrounding mineral conditions affect the surface processes and how the molecular and physicochemical inorganic aspects drive the prebiotic chemistry process.

In primitive Earth studies, special attention must be paid to inorganics interacting with organics [19] under different environmental conditions. Moreover, on terrestrial planets, such as Mars [20] and primitive Earth, a lack of an ozone layer in the first stages was possible, with a strong ultraviolet (UV) irradiation acting as a potential driving force for chemical activation [21,22,23,24,25]. Therefore, the role of mineral surfaces [26] would be crucial for studies on the origin of life in this context.

A detailed study of the pyrite UV oxidation process and reactivity of molecule/pyrite systems at the molecular level is fundamental to understanding the chemistry of this mineral in a wide variety of environments. Oxidation studies have shown that pyrite is reactive under ambient conditions and oxidizes rapidly in air, forming ferric sulfate. Other possible oxidation products including Fe-hydroxide or Fe-oxyhydroxide, monosulfide, disulfide and polysulfide are also formed. The formation of these oxidation products depends on the conditions and duration of the oxidation process. However, little is known about the surface chemistry of pyrite under vacuum conditions (simulated absence of oxygen), and few studies concerning oxidation, hydration and desulfurization reactions on pyrite surfaces have been reported [27,28]. The adsorption of amino acids on the surface of pyrite has been studied experimentally and theoretically [29,30]. The surface of pyrite has a rich chemistry due to the surface defect sites, where either iron or sulfur ions are exposed and can serve as adsorption sites for various ionic species. It has been reported by our group that sulfur enrichment drives molecular adsorption on pyrite, and the pyrite surface structure could dictate its molecular adsorption and reactivity properties, having relevant implications for prebiotic chemistry surface reactions. It is important to explore the role-played by the mineral surfaces related to the prebiotic chemistry processes, such as molecular adsorption and geochemistry, and therefore, we focused our study on investigating the possible role played by mineral surface reactivity. Our recent experiments have suggested that amino acids and small peptides can adsorb onto the mineral surfaces, depending on their structures, which are affected by the surroundings or surface pretreatment conditions. These studies provided some new insights into the adsorption process on surfaces. This strategy is designed to be able to evaluate how diverse environments favour or inhibit molecular adsorption and the crucial role played by mineral surfaces chemically driving amino-acids interactions. Even if amino acids only provide an interesting starting point for studying mineral—biomolecule interactions, small peptides could be a good way to achieve molecular complexity on surfaces.

**Figure 1:**
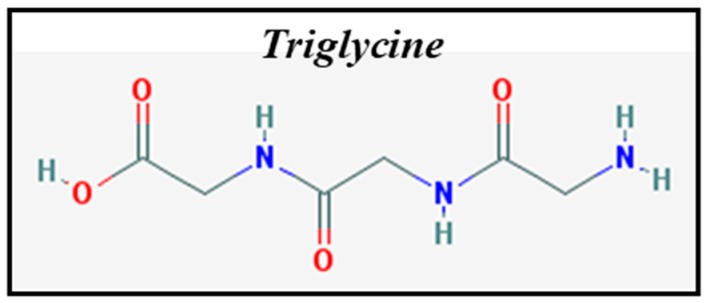
Chemical structure of triglycine.

Triglycine (Figure 1) is an amino-acid trimer formed by three glycine molecules (Gly-Gly-Gly). The glycine unit is the simplest possible amino acid. Triglycine is used as a model peptide for studies of the physicochemical parameters and molecular associations of small peptides, which can be used as a simple model of more complex molecules such as proteins. Triglycine provides two different functional groups, either or both of which could potentially be involved in bonding to mineral surfaces. In addition, triglycine molecule increases molecular complexity from amino acids to peptides studies, in order to study the role play by the peptide bond and to identify components on surface interaction, which has not been previously described.

In this context, we studied the pyrite oxidation resulting from UV irradiation and adsorption of the tripeptide triglycine (Figure 1) on pyrite by evaluating the interactions under different environmental conditions. It is crucial to compare experiments performed in the presence of water to others conducted under strictly ultra-high vacuum (UHV) conditions (simulated absence of oxygen and in-situ molecular deposition by evaporation in UHV), allowing for the relevant impact of these different conditions on the surface to be examined. Our surface science studies aim at a chemical characterization of the surface, investigating the strategic binding sites and the environmental conditions that are more favorable for increasing the probability of adsorption of molecular species and their subsequent concentration on the surface. Therefore, we report the results of a spectroscopic study of X-ray photoemission spectroscopy (XPS) data, obtained for pyrite under UV irradiation treatment and triglycine adsorption on a natural pyrite surface before and after UV treatment. Furthermore, we discuss spectroscopic evidence of different molecular adsorption mechanisms on the surface of pyrite constrained by oxidization (UV and/or water) or under ultra-high vacuum conditions.

## 2. Materials and Methods

Ultra-high vacuum conditions: The pyrite single crystal sample (provided by Surface Preparation Laboratory) was transferred to an ultra-high vacuum (UHV) chamber with a base pressure of 3 × 10^−10^ mbar. The pyrite sample was cleaned by repeated cycles of Ar^+^ ion sputtering at 800 eV and annealing at 595 K. Surface cleanliness was monitored using XPS. The pyrite sample was never overheated beyond 600 K to avoid thermal decomposition. Triglycine (NH_2_-CH_2_-CO-NH-CH_2_-CO-NH-CH_2_-COOH, purity ≥ 98%) powder was purchased from Sigma-Aldrich (Missouri, US) and used without further purification. A home-built molecular doser was employed to sublimate the tripeptide molecule in UHV conditions. The doser contained a tantalum envelope holding the chemical and a thermocouple facing the sample material. The tantalum envelope was heated by a direct current. A K-type thermocouple was attached to the bag on the outside, close to the chemical, to probe its temperature. Before sublimation, the triglycine was outgassed and then heated to 380 K and exposed to the pyrite crystal; in situ molecular deposition was done by evaporation under UHV conditions (simulated absence of oxygen). During the sublimation, the main chamber pressure typically rose to 1 × 10^−9^ mbar.

Solution experiments: A pyrite surface (from the Navajún mine in Spain) previously cleaned and dried was immersed into 10 mL of tri-Gly solution (1 mM, pH of 6.0) for 30 min at 298 K with stirring. After the immobilization step (surface being immersed in the molecular solution), the pyrite surface sample (solid) was rinsed with MilliQ-water, dried by blowing compressed air, and then analysed under UHV conditions using an XPS technique.

UV irradiation at air conditions: Pyrite samples were cleaned three times in different solutions of 1 M of H_2_SO_4_, in water (Milli-Q grade), dried by blowing air and then the pyrite surface was exposed to UV radiation (200–400 nm) for 5 and 27 h. A 150 W water-cooled deuterium UV lamp (Hamamatsu C3150), placed perpendicular to the pyrite sample, was used to irradiate the sample. The UV radiation from the lamp entered the system through a quartz window. The UV light hit a beam splitter placed very close to the lamp, which allowed 88% of the radiation to pass through. The other 12% of the beam was reflected onto another quartz window, where a UV detector was placed that permitted the continuous monitoring of the incoming UV flux via a spectroradiometer (Bentham DMc150FC). After the beam splitter, we set a focusing lens to focus the beam on the surface. The irradiance spectrum of the deuterium lamp was a continuum that decreased for increasing photon wavelength; the UV flux measured at the sample position, obtained by integration of the irradiance over the 200–400 nm wavelength range, was 2.3 × 10^14^ photons/cm^2^ in the current system. XPS spectra of the samples were recorded after UV exposure in a different dedicated XPS chamber; XPS of the pristine clean pyrite surface was also recorded to get information about the surface before UV irradiation.

X-ray photoelectron spectroscopy analysis of the samples was carried out in an ultrahigh-vacuum chamber equipped with a hemispherical electron analyser and an Al Kα X-ray source (1486.6 eV) with an aperture of 7 mm × 20 mm. The base pressure in the chamber was 3 × 10^−10^ mbar, and the experiments were performed at room temperature. The peak decomposition in different components was shaped, after background subtraction, as a convolution of the Lorenztian and Gaussian curves. Binding energies were calibrated against the binding energy of the C 1s peak at 285.0 eV for the pyrite solution samples and against the S 2p peak at 162.8 eV for the pyrite (FeS_2_) under the UHV samples. We had not observed any beam radiation damage of the triglycine layer during the data acquisition.

## 3. Results and Discussion

### 3.1. Effect of Ultraviolet (UV) Radiation on the Pyrite Surface

**Figure 2:**
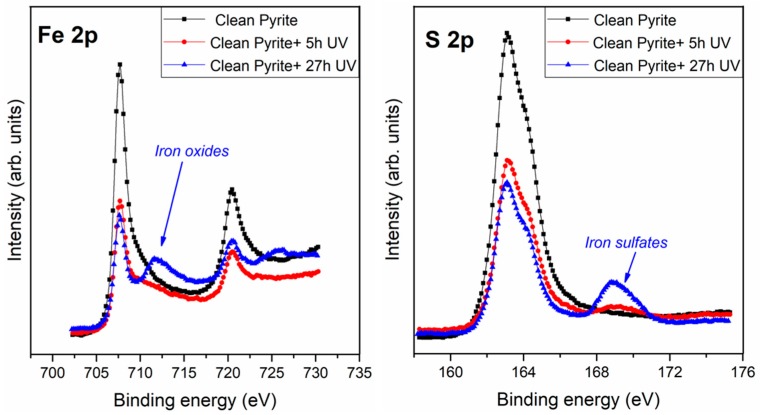
XPS photoemission spectra of Fe 2p, and S 2p core level peaks of the clean pyrite surface before and after 5 and 27 h of UV irradiation treatment.

Regarding the pyrite clean surface (see Figure 2), the sulfur peak showed three components: The main one at 162.8 eV, which corresponded to the S_2_^2−^, clean pyrite surface (FeS_2_, 77%), in agreement with the literature [31,32,33]; a second component at 164.7 eV assigned to a polysulfide species (S-S bonds, 12%) present in natural pyrite [33,34,35]; and a third component at 161.8 eV assigned to the S^2−^ (FeS, 11%). The iron spectrum showed a main component at 707.4 eV assigned to Fe^2+^ (clean iron sulphide, 69%), a second component at 708.9 eV assigned to Fe^2+^ (FeS + FeO, 24%) and a third component at 711.1 eV assigned to Fe^3+^ (Fe_2_O_3_, 7%) (see Table 1).

Once the surface had been exposed to UV irradiation for 5 and 27 h, the XPS spectra shows that all the peaks seen and identified in the non-irradiated pyrite surface, are also present in the pyrite UV irradiated for 5 and 27 h (see Table 1). In addition to these peaks, new peaks resulting from the UV irradiation were observed at 168.5 eV and at 712.9 eV, assigned to sulfate (SO_2_^4−^) species and to Fe^3+^ (Fe_2_SO_4_), respectively. It is noticeable that the proportions between these peaks changed significantly after 5 and 27 h of UV irradiation from 4–6% to 12–22% respectively (see Table 1).

UV irradiation has an effect on the pyrite surface even after a very short time, such as 5 h of exposure, confirmed by the appearance of the sulfate species and a remarkable increase in the oxidized species; furthermore, a higher increase in the oxides and sulfate species is shown at 27 h of exposure. Consequently, the pyrite surface is highly sensitive to chemical changes induced by UV irradiation, meaning that pyrite surface chemistry could be easily affected by environmental conditions and is highly reactive, which would be a necessary condition for a potentially effective catalyst.

**Figure 3:**
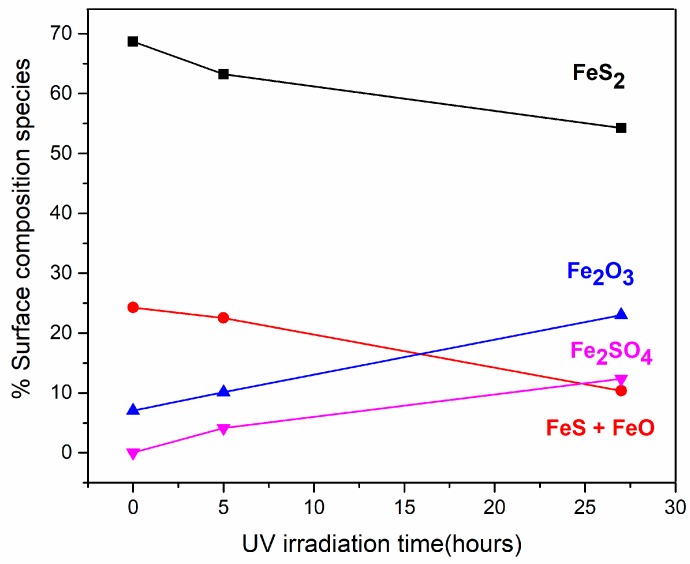
Fe and S surface species distribution along the UV irradiation process duration at 0, 5 and 27 h of UV irradiation of the surface.

The UV irradiation process of the surface over several hours (5 and 27 h) results in remarkable changes in the shape of the S 2p and Fe 2p spectrum (Figure 2). To clarify the chemical changes induced on the pyrite surface, the distributions of the S and Fe species during the UV irradiation process have been represented (see Figure 3).

The XPS data for the Fe species showed a decrease of the 707.3 eV signal (FeS_2_, black line) and the 709.1 eV signal (FeS + FeO, red line) after increasing the UV irradiation time. Finally, the Fe_2_O_3_ contribution at the 711.1 eV signal (blue line) increased, and a significant appearance of the signal from Fe_2_SO_4_ (pink line) at 712.9 eV, along the UV irradiation process, developed. This is in agreement with the sulfur XPS results. For the sulfur species, the 161.9 eV component assigned to the FeS + FeO species (red line) decreased, and the main component at 162.8 eV assigned to the FeS_2_ species (black line) decreased as well; there was the remarkable appearance of a new component assigned to the Fe_2_SO_4_ at 168.5 eV (pink line), which appeared due to the UV irradiation process, and the component at 164.6 eV assigned to polysulfides (S_n_^2−^) remained constant as expected.

In summary, the exposure of pyrite to longer UV irradiation processes (5 and 27 h) induced a decrease in the originally observed chemical species, assigned to phases disulfide (FeS_2_), monosulfide (FeS) and FeO (see Figure 3). The increased amount of Fe_2_O_3_ species was obtained because of the UV irradiation process during 5 h and 27 h, which also induced the appearance of the iron sulfates species. The pyrite UV irradiation process carried out the oxidation of Fe^2+^ into Fe^3+^, and the presence of Fe^3+^ favoured the oxidising process of Fe^2+^. Therefore, Fe^3+^ is a reaction product in the presence of oxygen (Fe^2+^ → Fe^3+^) as well as an oxidant reactive for pyrite (the presence of Fe^3+^ favour the oxidising process of Fe^2+^); in this sense, the oxidation of pyrite due to the UV irradiation treatment can be considered an autocatalytic process [27]. Pyrite is a good catalyst candidate since its surface is very reactive, even after a short time of <5 h of UV exposure. Furthermore, we are interested in the molecular interaction of short peptides from inert conditions under UHV conditions to more reactive conditions and molecular adsorption from solutions before and after UV pyrite surface treatment.

### 3.2. Interaction of Triglycine with Pyrite Surfaces under Several Conditions

#### 3.2.1. Interaction of Triglycine with Pyrite Surfaces by Molecular Evaporation Deposition under Ultra-High Vacuum Conditions

**Figure 4:**
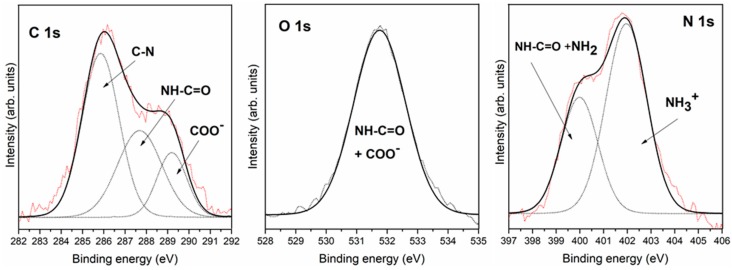
XPS spectra for triglycine adsorption on the pyrite surface from the gas phase under UHV conditions. These XPS spectra were obtained immediately after the triglycine adsorbed on the surface.

Triglycine adsorption on the pyrite surface under ultra-high vacuum conditions (absence of oxygen and in-situ molecular deposition by evaporation in UHV) was analysed by XPS, which is well suited to characterize the possible chemical changes in molecular interactions on surfaces due to its high sensitivity and non-destructive nature. The in situ XPS analysis of triglycine adsorption on the pyrite surface revealed the presence of carbon, oxygen and nitrogen on the pyrite surface under UHV conditions (see Figure 4). The best-fit curve for the C 1s peak was obtained using three components for the small peptide, triglycine, and the first carbon component had a binding energy (B.E.) of 285.8 eV and was attributed to the C-N group [36,37]; the second component was observed at 287.7 eV due to the amide group (-NH-CO-), and the presence of a third carbon component at 289.2 eV was assigned to the COOH or COO^−^ groups [38]. The intensity ratio of the three components was 3:2:1 (CN: -NH-CO-:COO^−^ or COOH), which is in close agreement with the chemical formula of the molecule. The O 1s peak was observed at 531.8 eV and was attributed to one component, the oxygen in the COO^−^ groups [31,32,33,34,39] and the C=O of the amide group. The best-fit curve of the N 1s peak consists of two components centred at the binding energies of 400.0 and 402.0 eV, which were assigned to the NH_2_ + -NH-CO- and NH_3_^+^ functional groups, respectively [27,34,35] (see Figure 1 for the triglycine molecule). XPS demonstrates successful adsorption of the intact molecule on the pyrite surface.

The presence of the NH_3_^+^ functional group was only observed when the molecular adsorption occurred under UHV conditions, which favours the presence of the NH_2_ and NH_3_^+^ functional groups, for cystine molecules, as previously reported by our group [30]. UHV conditions facilitate the formation of a diversity of chemical species. Because the anionic form of amino acids is the predominant form observed when these compounds adsorb onto surfaces, it is remarkable that NH_3_^+^ was observed on the surface of pyrite only under UHV conditions. The presence of sulfur vacancies on the surface may rationalize the adsorption of cations under UHV conditions because they are compensated for [40,41], whereas under solution conditions, sulfur vacancies are readily passivated by water molecules, and the presence of iron oxides makes it more likely that only the anionic form of the molecule will be present. Surface vacancies on pyrite could affect the chemical form in which the molecule is adsorbed, determining the molecular chemistry on the surface. We focused our studies to confirm this fact for more complex molecules than amino acids, such as the small peptide, triglycine.

#### 3.2.2. Interaction of Triglycine Solution Adsorption on the Pyrite Surface

**Figure 5:**
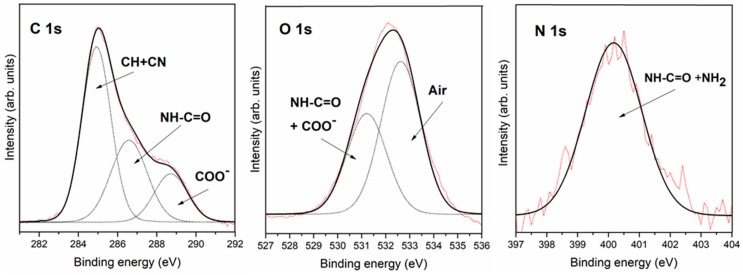
XPS photoemission spectra of the C 1s, O 1s and N 1s core-level peaks of triglycine adsorbed on the pyrite surface from solution 1 mM. Experimental core-level spectra (red) and the fitting results for all the components (black) and individual components (dotted lines).

Figure 5 shows the XPS spectra for the triglycine adsorption on a pyrite surface from a solution. The C 1s peak shows three contributions at 284.9 eV, 286.6 eV and 288.7 eV, which were assigned to C-H groups with a contribution from contamination during sample preparation in air instead of under UHV conditions and CN groups, to the amide group (-NH-CO-) and to the carboxylic groups or carboxylate groups (COO^−^), respectively [30]. Regarding the O 1s peak, we fitted the experimental data points using two components. The first component occurred at a binding energy of 531.2 eV, which was assigned to the two equivalent oxygen atoms belonging to the so-called resonant state of the deprotonated carboxylic group (carboxylate group) and to the amide group (-NH-CO-) [31,32,33,34,39], and the second component appears at 532.2 eV, but this component is a contribution from contamination during sample preparation in air instead of under UHV conditions. The N 1s core level peak presented a single component centred at 400.2 eV, which was assigned to the NH_2_ species and to the amide group (-NH-CO-), indicating that anions in the solution were the only adsorption species present on the pyrite surface. The molecule successfully adsorbed on the pyrite surface.

#### 3.2.3. Interaction of Triglycine Solution Adsorption on the UV Oxidized Pyrite Surface

Once we had studied and characterized pyrite surface oxidation under UV exposure conditions, we turned to study triglycine peptide affinity adsorbed on the pyrite oxidized surface. Therefore, triglycine adsorption on the pyrite surface was analysed by XPS, which is well suited to characterize the possible chemical changes in molecular interactions on surfaces due to its high sensitivity and non-destructive nature.

XPS analysis of triglycine adsorption on the UV oxidized pyrite surface revealed the presence of carbon, oxygen and nitrogen on the pyrite surface. The best-fit curve for the C 1s peak was obtained using three components for the small peptide. The first carbon component had a binding energy (B.E.) of 284.9 eV assigned to the air contribution and CH, a second component at 286.5 eV was attributed to the C-N group [31,32] and the amide group (-NH-CO-), whereas the third component was observed at 288.5 eV and was assigned to the COOH or COO^−^ groups [33]. For the O 1s, a peak was observed at 531.4 eV and was attributed to one component; the oxygen in the COO^−^ groups [31,32,33,34,39], and amide group, and the second component at 532.5 eV was assigned to the air contribution. The best-fit curve of the N 1s peak consisted of only one component centred at the binding energy of 399.8 eV, which was assigned to the NH_2_ and the amide group (-NH-CO-) [33,34,35].

**Figure 6:**
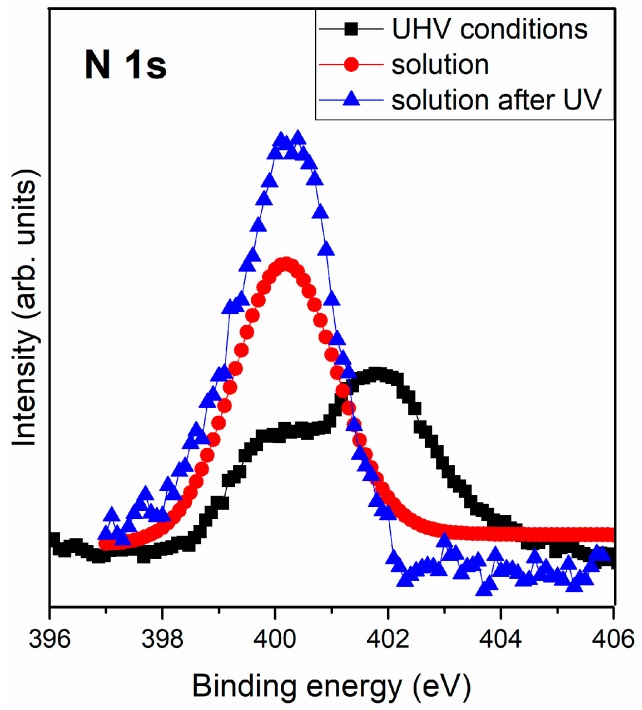
XPS photoemission spectra of N 1s core level peak of triglycine adsorbed from the gas phase under anoxic conditions and from solution before and after pyrite surface UV irradiation exposure.

Therefore, the triglycine peptide was successfully adsorbed in three cases: UHV conditions and from a solution before and after UV pyrite surface treatment. Remarkable differences were shown based on the nitrogen signal: two chemical forms were observed under UHV conditions, i.e., NH_2_ and NH_3_^+^, whereas only the NH_2_ was detected when adsorbing the molecule from solution. Figure 6 shows nitrogen differences from the three different molecular adsorption conditions. The XPS nitrogen signal is a molecular fingerprint on its surface, and a higher intensity of the signal means a higher concentration of molecules on the surface; therefore, as has been previously explained, UV irradiation favours molecular adsorption of triglycine on the pyrite surface (see Figure 6).

A summary of the XPS results, C1 s, N1 s and O1 s core-level peaks and assignments in their components for triglycine/pyrite under UHV conditions, molecular immobilization from solution and after UV surface irradiation treatment, are shown in Table 2.

A comparison study of triglycine adsorption on the pyrite surface shows the presence of the NH_2_ and NH_3_^+^ functional groups, under UHV conditions, but nevertheless, more molecules accumulated on the surface when molecular adsorption was conducted from a solution and was even higher if the pyrite surface had been previously exposed to UV irradiation (see the nitrogen signal from Figure 6). The fact that the surface had been oxidized, inferred different properties. UV irradiation makes the pyrite surface more reactive and enables it to accumulate more molecules; this fact would be a helpful condition to allow for reactions between molecules. Therefore, these results are in agreement with the Bernal theory that mineral surface adsorption could help to overcome the problem of an extremely dilute concentration of amino acids in prebiotic oceans. Minerals can be potential surfaces to adsorb molecules, and furthermore, environmental conditions could be the determining factor to increase the probability of molecular reactions.

From these experiments, we have calculated triglycine molecules adsorbed on the surface of the pyrite during different experimental conditions. Calculations of the nitrogen/sulfur ratio for each experimental case, taking in account the experimental area (I) and the atomic sensitivity factor (S) for X-ray source for each element has been done. The equation applied is the following:

n_1_/n_2_ = (I_1_/S_1_)/(I_2_/S_2_) (1)

N/N = (I_Nitrogen_/S_Nitrogen_)/(I_Sulfur_/S_Sulfur_) (2)

Then, the ratio N/S for UHV conditions is the lowest with a value of 0.06, for solution conditions is 0.12 and for solution after UV conditions is the highest ratio with a value 0.18, meaning that immobilization of the triglycine molecules from solution after UV conditions helps to concentrate molecules on the surface.

The pyrite surface is reactive to environmental conditions, such as UHV conditions, water solutions, UV irradiation and chemical changes induced on the surface, which modify the molecular interactions; furthermore, pyrite surfaces show an affinity for adsorption of molecules, including small peptides. The pyrite surface can be proposed as a good reservoir to concentrate molecules and to have the potential to react when the environmental conditions are optimal. The ability to respond to environmental conditions and its molecular affinity have positioned pyrite as a good candidate for being a surface promotor of catalytic reactions in the prebiotic chemistry context. Chemically, the fact that iron sulfide surfaces contain at least two types of surface functional groups has some interesting implications, such as the possibility of binding species that are chemically different and the subsequent interactions, including electron transfer, between the co-adsorbates that may produce products that would be difficult to form on a surface with a single surface site.

Molecular adsorption from solution conditions was slightly more favorable, concentrating a larger number of molecules on the surface compared to under UHV conditions (see the nitrogen comparison study, Figure 6), even though only anionic chemical species were present on the surface. In solution, the carbon and oxygen peaks showed atmospheric and water contributions on the surface of pyrite, producing a stronger overall spectral signal than that observed under UHV conditions. Hence, the triglycine molecules are adsorbed only in their more favorable anionic form. On the other hand, under UHV conditions, in which sulfur anion vacancies are not compensated for, the zwitterionic and anionic chemical species coexist on the pyrite surface.

UV exposure rapidly changes pyrite surface species, and furthermore, UV exposure has critical implications for the adsorption of triglycine molecules onto the pyrite surface and changing molecular chemical functionalities is expected to affect its molecular reactivity between different chemical species; therefore, it is relevant to study conditions which may be encountered in various natural environments.

In summary, this spectroscopic analysis demonstrates the presence of oxide compounds as well as new sulfur species on the surface of pyrite due to the UV irradiation and indicates that triglycine adsorbs on the pyrite surface in its anionic form when adsorbed from solution. Furthermore, UV is a relevant parameter to increase molecular concentration on pyrite surfaces. The findings and their implications are relevant in the prebiotic chemistry research field, showing that environmental conditions drive the appearance of new species on surfaces, which modify the molecular interactions. Furthermore, a UV irradiation of mineral surfaces may help to concentrate such compounds for potential catalytic reactions on surfaces to occur.

## 4. Conclusions

We have performed the first spectroscopic characterization of triglycine adsorption on a UV irradiated pyrite surface. XPS analysis was employed to efficiently explore the molecular adsorption, to understand surface chemistry, and finally, to describe the critical influence of the different environmental conditions in the small peptides-pyrite system. We demonstrated how pyrite minerals can concentrate and act as adsorption substrates for small peptides during wetting conditions and even under inert UHV conditions. The successful adsorption of several molecules from amino acids to small peptides under a wide range of experimental conditions indicates the potential of pyrite as a catalyst under different environmental conditions. The pyrite surface offers the possibility of binding species that are chemically different as a multiple-site surface, and therefore, the co-adsorbates may produce products that would be difficult to form on a surface with a single surface site. These studies could therefore shed light onto prebiotic chemistry reactions.

Mineral surfaces may play a relevant role to concentrate molecules, even under extremely diverse environments (UHV, solution, UV), which does not inhibit molecule/mineral surface interaction. Therefore, minerals could act as a molecular surface concentrator to overcome the controversy matter from “dilute prebiotic soup”. Mimicking the complex prebiotic geochemical environment is still an enormous challenge; nevertheless, different environmental conditions could help to discriminate under which conditions molecule/mineral interactions would or would not be negligible.

## Figures and Tables

**Table 1:** A Comparison study of Fe 2p and S 2p core-level peaks, deconvolution and ratios of their components peaks of the clean pyrite surface before and after 5 and 27 h of UV irradiation treatment.

	Clean Pyrite		Clean Pyrite + 5 h UV		Clean Pyrite + 27 h UV		Assignment
	BE (eV)	%	BE (eV)	%	BE (eV)	%	
***Fe 2p***	707.3	69	707.3	63	707.3	54	Fe^2+^ (FeS_2_)
	708.9	24	709.1	23	708.9	10	Fe^2+^ (FeS + FeO)
	711.1	7	711.1	10	711.1	23	Fe^3+^ (Fe_2_O_3_)
			712.9	4	712.9	12	Fe^3+^ (Fe_2_SO_4_)
***S 2p***	161.8	11	161.9	10	161.9	6	S^2−^ (FeS)
	162.8	77	162.8	71	162.8	60	S_2_^2−^ (FeS_2_)
	164.7	12	164.6	13	164.6	12	S_n_^2−^ (2 ≤ n ≤ 8)
			168.5	6	168.5	22	SO_2_^4−^ (sulfate)

**Table 2:** A Comparison study of C 1s, N 1s and O 1s core-level peaks and deconvolution of their components for the triglycine/pyrite system under different environmental conditions (UHV, solution and solution after UV).

	UHV Conditions		Solution		Solution after UV	
	BE (eV)	Assignment	BE (eV)	Assignment	BE (eV)	Assignment
***C 1s***	285.8	C-N	284.9	C-H + air	284.9	C-H + air
	287.7	-NH-CO-	286.6	C-N + -NH-CO-	286.5	C-N + -NH-CO-
	289.2	COOH or COO^−^	288.7	COOH or COO^−^	288.5	COOH or COO^−^
***O 1s***	531.8	COO^−^ + -NH-CO-	531.2	COO^−^ + -NH-CO-	531.4	COO^−^ + -NH-CO-
			532.2	air	532.5	air
***N 1s***	400.0	NH_2_ + -NH-CO-	400.2	NH_2_ + -NH-CO-	399.8	NH_2_ + -NH-CO-
	402.0	NH_3_^+^				

## References

[1] Cleaves H.J., Scott A.M., Hill F.C., Leszczynski J., Sahai N., Hazen R. (2012). Mineral-organic interfacial processes: Potential roles in the origins of life. Chem. Soc. Rev..

[2] Colin-Garcia M., Heredia A., Negron-Mendoza A., Ramos-Bernal S. (2012). Organics-Minerals Interactions and the Origin of Life.

[3] Bernal J.D. (1951). The Physical Basis of Life.

[4] Schoonen M., Smirnov A., Cohn C. (2004). A perspective on the Role of Minerals in Prebiotic Synthesis. Ambio.

[5] Lambert J.F. (2008). Adsorption and polymerization of amino acids on mineral surfaces: A review. Orig. Life Evol. Biospheres.

[6] Rosso K.M., Becker U., Hochella M.F. (1999). Atomically resolved electronic structure of pyrite {100} surfaces: An experimental and theoretical investigation with implications for reactivity. Am. Mineral..

[7] Liu T., Temprano I., Jenkins S.J., King D.A., Driver S.M. (2012). Nitrogen adsorption and desorption at iron pyrite FeS_2_ 100 surfaces. Phys. Chem. Chem. Phys..

[8] Liu T., Temprano I., Jenkins S.J., King D.A., Driver S.M. (2013). Low Temperature Synthesis of NH3 from Atomic N and H at the surfaces of FeS2{100} Crystals. J. Phys. Chem..

[9] Saladino R., Neri V., Crestini C., Costanzo G., Graciotti M., Di Mauro E. (2008). Synthesis and degradation of nucleic acid components by formamide and iron sulfur minerals. J. Am. Chem. Soc..

[10] Lahav N., Chang S. (1976). The Possible Role of Solid Surface Area in Condensation Reactions during Chemical Evolution: Reevaluation. J. Mol. Evol..

[11] Huber C., Wächtershäuser G. (1998). Peptides by Activation of Amino Acids with CO on (Ni,Fe)S Surfaces: Implications for the Origin of Life. Science.

[12] Hazen R.M., Sverjensky D.A. (2010). Mineral surfaces, geochemical complexities, and the origins of life. Cold Spring Harb. Perspect. Boil..

[13] Ferris J.P., Hill A.R., Liu R., Orgel L.E. (1996). Synthesis of long prebiotic oligomers on mineral surfaces. Nature.

[14] Zaia D.A.M. (2004). A review of adsorption of amino acids on minerals: Was it important for origin of life?. Amino Acids.

[15] Rode B.M., Son H.L., Suwannachot Y., Bujdak J. (1999). The combination of salt induced peptide formation reaction and clay catalysis: A way to higher peptides under primitive Earth conditions. Orig. Life Evol. Biospheres.

[16] Fornaro T., Brucato J.R., Feuillie C., Sverjensky D.A., Hazen R.M., Brunetto R., D’Amore M., Barone V. (2018). Binding of Nucleic Acid Components to the Serpentinite-hosted Hydrothermal Mineral Brucite. Astrobiology.

[17] Powner M.W., Gerland B., Sutherland J.D. (2009). Synthesis of activated pyrimide ribonucleotides in prebiotically plausible conditions. Nature.

[18] Scappini F., Casadei F., Zamboni R., Franchi M., Gallori E., Monti S. (2004). Protective effect of clay minerals on adsorbed nucleic acid against UV radiation: Possible role in the origin of life. Int. J. Astrobiol..

[19] Degens E.T. (1989). Perspectives on Biogeochemistry.

[20] Patel M.R., Bérces A., Kerékgyárto T., Rontó Gy., Lammer H., Zarnecki J.C. (2004). Annual solar UV exposure and biological effective dose rates on the Martian surface. Adv. Space Res..

[21] Cockell C.S. (2000). Ultraviolet radiation and the photobiology of earth’s oceans. Orig. Life Evol. Biospheres.

[22] Horneck G. (2007). Complete Course in Astrobiology.

[23] Dos Santos R., Patel M., Cuadros J., Martins Z. (2016). Influence of mineralogy on the preservation of amino acids under simulated Mars conditions. Icarus.

[24] Fornaro T., Boosman A., Brucato J.R., ten Kate I.L., Siljeström S., Poggiali G., Steele A., Hazen R.M. (2018). UV Irradiation of Biomarkers Adsorbed on Minerals under Martian-like Conditions: Hints for Life Detection on Mars. Icarus.

[25] Poch O., Jaber M., Stalport F., Nowak S., Georgelin T., Lambert J.-F., Szopa C., Coll P. (2015). Effect of Nontronite Smectite Clay on the Chemical Evolution of Several Organic Molecules under Simulated Martian Surface Ultraviolet Radiation Conditions. Astrobiology.

[26] Cleaves II H.J., Lazcano A., Ledesma Mateos I., Negrón-Mendoza A., Peretó J., Silva E. (2014). Herrera’s Plasmogenia and Other Collected Works. Early Writting on the Experimental Study of the Origin of Life.

[27] Eggleston C.M., Ehrhardt J.J., Stumm W. (1996). Surface structural controls on pyrite oxidation kinetics: An XPS-UPS, STM, and modeling study. Am. Mineral..

[28] Karthe S., Szargan R., Suoninen E. (1993). Oxidation of pyrite surfaces: A photoelectron spectroscopic study. Appl. Surface Sci..

[29] De la Cruz-López A., Del Ángel-Meraz E., Colín-García M., Ramos-Bernal S., Negrón-Mendoza A., Heredia A. (2017). Ultraviolet irradiation of glycine in presence of pyrite as a model of chemical evolution: An experimental and molecular modelling approach. Int. J. Astrobiol..

[30] Sanchez-Arenillas M., Mateo-Marti E. (2015). Spectroscopic study of cystine adsorption on pyrite surface: From vacuum to solution conditions. Chem. Phys..

[31] Uvdal K., Bodö P., Ihs A., Lieberg B., Salaneck W.R. (1990). X-ray photoelectron and infrared spectroscopy of clycine adsorbed upon copper. J. Colloid Interface Sci..

[32] Uvdal K., Bodö P., Lieberg B. (1992). L-cysteine adsorbed on gold and copper: An X-ray photoelectron spectroscopy study. J. Colloid Interface Sci..

[33] Mateo-Marti E., Rogero C., Briones C., Martín-Gago J.A. (2007). Does peptides nucleic acids on pyrite surface form self-assembled monolayers?. Surf. Sci..

[34] Sanchez-Arenillas M., Mateo-Marti E. (2016). Pyrite surface environment drives molecular adsorption: Cystine on pyrite (100) investigated by X-ray photoemission spectroscopy and low energy electron diffraction. Phys. Chem. Chem. Phys..

[35] Sanchez-Arenillas M., Galvez-Martinez S., Mateo-Marti E. (2017). Sulfur amino acids and alanine on pyrite (100) by X-ray photoemission spectroscopy: Surface or molecular role?. Appl. Surf. Sci..

[36] Cavalleri O., Gonella G., Terreni S., Vignolo M., Floreano L., Morgante A., Canepa M., Rolandi R. (2004). High resolution X-ray photoelectron spectroscopy of L-cysteine self-assembled films. Phys. Chem. Chem. Phys..

[37] Gonella G., Terreni S., Cvetko D., Cossaro A., Mattera L., Cavalleri O., Rolandi R., Morgante A., Floreano L., Canepa M. (2005). Ultrahigh vacuum deposition of L-cysteine on Au (110) studied by high-resolution X-ray photoemission: From early stages of adsorption to molecular organization. J. Phys. Chem. B.

[38] Fischer S., Papageorgiou A.C., Marschall M., Reichert J., Diller K., Klappenberger F., Allegretti F., Nefedov A., Wöll C., Barth J.V. (2012). L-Cysteine on Ag (111): A combined STM and X-ray spectroscopy study of anchorage and deprotonation. J. Phys. Chem. C.

[39] Clark D.T., Peeling J., Colling L. (1976). An experimental and theoretical investigation of the core level spectra of a series of amino acids, dipeptides and polypeptides. Biochim. Biophys. Acta.

[40] Andersson K., Nyberg M., Ogasawara H., Nordlund D., Kendelewicz T., Doyle C.S., Brown G.E., Pettersson L.G.M., Nilsson A. (2004). Experimental and theoretical characterization of the structure of defects at the pyrite FeS_2_ (100) surface. Phys. Rev. B.

[41] Andersson K.J., Ogasawara H., Nordlund D., Brown G.E., Nilsson A. (2014). Preparation, Structure, and Orientation of Pyrite FeS_2_ {100} Surfaces: Anisotropy, Sulfur Monomers, Dimer Vacancies, and a Possible FeS Surface Phase. J. Phys. Chem. C.

